# Comparative Evaluation of Flavor and Sensory Quality of Coffee Pulp Wines

**DOI:** 10.3390/molecules29133060

**Published:** 2024-06-27

**Authors:** Rongsuo Hu, Fei Xu, Liyan Zhao, Wenjiang Dong, Xingyuan Xiao, Xiao Chen

**Affiliations:** 1 College of Food Science and Technology, Nanjing Agricultural University, Nanjing 210095, China; hnhrs@126.com (R.H.); chenxiao@njau.edu.cn (X.C.); 2 Spice and Beverage Research Institute, Chinese Academy of Tropical Agricultural Sciences (CATAS), Wanning 571533, China; xufei_0302054@163.com; 3 Key Laboratory of Processing Suitability and Quality Control of the Special Tropical Crops of Hainan Province, Wanning 571533, China; 4 College of Tropical Crops, Yunnan Agriculture University, Pu’er 665000, China; X15758390485@126.com

**Keywords:** coffee, coffee pulp wine, sensory characteristic, flavor profile

## Abstract

Coffee pulp wines were produced through the mixed fermentation of *Saccharomyces cerevisiae*, and the flavor and sensory characteristics were comparatively evaluated. A total of 87 volatile components were identified from five coffee pulp wines, of which 68 were present in all samples, accounting for over 99% of the total concentration. The sample fermented contained significantly higher levels of volatile metabolites (56.80 mg/g). Alcohols (22 species) and esters (26 species) were the main flavor components, with the contents accounting for 56.45 ± 3.93% and 31.18 ± 4.24%, respectively, of the total. Furthermore, 14 characteristic components were identified as potential odor-active compounds, contributing to sweet and floral apple brandy flavor. Although the characteristic components are similar, the difference in the content makes the overall sensory evaluation of the samples different. The samples formed by fermentation of four strains, which obtained the highest score (86.46 ± 0.36) in sensory evaluation, were further interpreted and demonstrated through the Mantel test. The results of the component analysis were effectively distinguished by OPLS-DA and PCA, and this validation was supported by sensory evaluation. The research results provided a technical reference for the production of coffee pulp wines.

## 1. Introduction

Coffee pulp was a by-product of primary processing, and Esquivel provided a definition of coffee pulp as a composite of exocarp and mesocarp [1]. It constitutes approximately 40–50% of the total weight of the coffee cherry [2], resulting in an annual production of up to 9.4 million tons [3]. Regrettably, coffee pulps had not received adequate attention and were commonly regarded as agricultural waste. The high sugar concentration in coffee pulp results in the production of unpleasant odors and wastewater during the process of fermentation and degradation. It may lead to the acidification of soil and rivers, seriously threatening the ecological environment and water system [4]. 

Coffee pulp exhibits a high nutrient content and holds promising potential for advancement and utilization in relation to its nutrient composition and biological properties. The nutrient profile of coffee pulp encompasses carbohydrates (45~89% of the dry matter), proteins (4~12% of the dry matter), lipids (1~2% of the dry matter), and minerals (6~10% of the dry matter) [5]. Additionally, the coffee pulp is abundant in polyphenolic compounds, notably chlorogenic acid, hydroxycinnamic acid, anthocyanins, flavan-3-ols, and flavanols [6], which have favorable preventive and therapeutic effects on chronic diseases [7]. 

In recent years, researchers have taken great interest in the development and utilization of coffee pulp. The addition of coffee pulp powders in the production of bread and biscuits has resulted in products that increase the level of dietary fiber [8,9]. Moreover, coffee pulp can be incorporated into various food items owing to its inherent sweetness, such as jam, juice, fruit puree, jelly, and cold beverages [10,11,12]. Additionally, coffee pulp can be transformed into coffee pulp tea [13]. Notably, the European Union has designated coffee pulp as a “new food” [14]. 

Coffee pulp wine is a novel fermented beverage characterized by a 7–10° alcohol content and favorable taste. Due to the relatively low concentration of flavor compounds inherent in the coffee pulp, the main source of flavor in all coffee pulp wines comes from the fermentation of yeast. Therefore, the crucial role of Saccharomyces cerevisiae in shaping the distinctive flavor of coffee pulp wine was emphasized [15]. To address the fermentation limitation of monotonous strain and enhance the diversity of flavors [16], the fermentation approach of a multi-strain composite was employed to augment the sensory complexity of coffee pulp wines.

Sensory characteristics of wine can be considered as one of the key factors in the acceptance of the wine [17]. The quality of wine is defined by several factors, including aroma, appearance, mouthfeel, and taste [18]. Characterizing the sensory properties of a set of wines with a trained panel and a descriptive analysis procedure, as commonly carried out by many wine research groups worldwide, is highly valuable [19]. However, new sensory analysis evaluation methods have been developed that allow untrained panelists to describe products using their own vocabulary [20]. Among these methods, Free-Comment (FC) has been used in several studies, for example in French wines [21].

Here, we hypothesize that the fermentation of a multi-strain composite has different effects on flavor and sensory properties, and these differences can be effectively detected using statistical models. Therefore, we evaluated the flavor and sensory quality of coffee pulp wines fermented by different strain combinations to determine the flavor characteristics of each strain combination and to determine the optimal fermentation combination to provide a theoretical basis for subsequent research and coffee pulp wine production.

## 2. Results and Discussion

### 2.1. HS-SPME-GC—MS Analysis of Volatile Components

**Figure 1:**
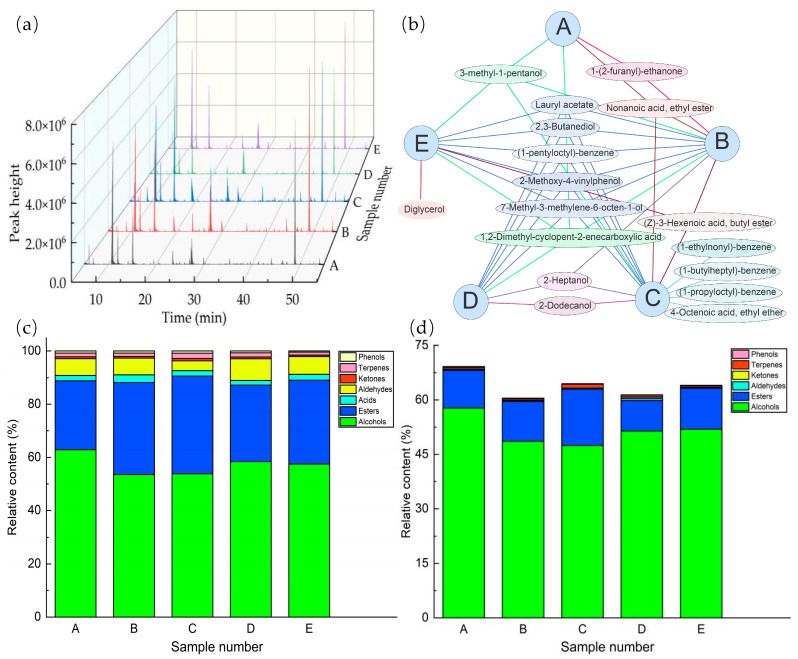
Volatile components analysis. (**a**) Ion flow chart of samples, (**b**) Venn network diagram, (**c**) classes and relative content analysis of volatile compounds, and (**d**) classes and relative content analysis of characteristic compounds.

The volatile component was one of the important indicators to evaluate the quality of coffee pulp wines. Volatile compounds and ion flow charts are shown in Figure 1 and Table 1. Two methods were used to identify volatile compounds (retention index and spectrum library identification). The retention index method was a very reliable method that was only affected by the chromatographic column and had little to do with the detection procedure. It was used in many foods, such as yogurt [22] and faba beans [23]. The identification of compounds could be completed by spectral library identification and retention index alignment, so it was not necessary to carry out standard substance verification.

A total of 79 volatile components of seven categories were detected from five samples of coffee pulp wines. The compound numbers of the five samples were 67, 73, 76, 70, and 71, respectively (from sample A to sample E). In total, 62 common compounds were present in five samples. More interestingly, the relative content of common compounds accounted for 99.20 ± 0.36% of the total content. 

Alcohols (22 species) and esters (25 species) were the main flavor components. The other compounds were 10 phenols, 7 terpenes, 6 ketones, 6 acids, and 3 aldehydes, respectively. In other fermented fruit wines, alcohols and esters are also the two most abundant components [24]. The relative contents of alcohol compounds were 62.91%, 53.51%, 53.83%, 58.36%, and 57.45%, respectively. The relative contents of ester compounds were 25.96%, 34.68%, 36.78%, 28.77%, and 31.60%, respectively. The relative content of alcohols and esters accounted for 88.76 ± 1.28% of the total content of volatile compounds (88.86%, 88.19%, 90.61%, 87.12%, and 89.04%, respectively). In conclusion, although the composition and content of alcohols and esters in the five samples were different, while the contents of the total volatile components were also different, the ratio of the content of alcohol and ester to the total content was relatively stable.

Figure 1a was the chromatogram of the five samples, which intuitively showed that the five samples had similar compounds but had some differences in content. It was possible that the samples were fermented by different combinations of four yeast strains. Figure 1b serves as a valuable addition to Figure 1a. It elucidated the affiliation of 17 volatile compounds that were non-common compounds in the samples. There were eight compounds in four samples, two compounds in three samples, two compounds in two samples, and five compounds in one sample. For instance, 2-methoxy-4vinylphenol existed in four samples (sample B, sample C, sample D, and sample E), whereas (1-butylheptyl)-benzene exclusively manifested in sample C. Figure 1c shows the differences in the classes and relative content of volatile compounds. It was also intuitive to show the classification of compounds, such as esters and alcohols, which were the main compounds. Figure 1d shows the differences in classes and relative content of the characteristic components. The characteristic compounds belonged to six different classes, and the relative contents were 69.88% (sample A), 61.05% (sample B), 65.87% (sample C), 62.21% (sample D), and 64.82% (sample E). The content of volatile compounds and characteristic compounds varied significantly between the samples.

### 2.2. Identification Analysis of Characteristic Components

The technique of ROAV to determine whether it was a characteristic component has been extensively used [25,26]. The characteristic components are shown in Table 2. A total of 14 characteristic flavor components were identified. There are six key flavor components (ROAV ≥ 1), which play a key role in the formation of flavor in coffee pulp wines, making the wine present a sweet and floral apple brandy flavor. There are eight important flavor components that play an important role in modifying the flavor of coffee pulp wines, making the wine have slight honey, spice, fruity, and smokey flavors. Meanwhile, it was also known that there were differences in ROAVs among different samples, which made the coffee pulp wines show their own unique flavor.

#### 2.2.1. Alcohol Compounds Analysis

Alcohols were the main body of coffee pulp wines. The presence of higher alcohols made the body softer and fuller. Alcohols mainly came from the conversion of sugars in the aerobic environment during fermentation and the conversion of amino acids under anaerobic conditions. A small number of higher alcohols could be produced by yeast reducing the corresponding aldehydes.

Twenty-two higher alcohols were identified in five samples. There are four characteristic components, among which one is the key flavor substance (phenylethyl alcohol). The content of alcohols was higher, but because the threshold was also higher, alcohols often did not affect the flavor of coffee pulp wines. Benzyl alcohol gave the samples a fresh bread and a sweet rose-like aroma [27].

#### 2.2.2. Ester Compounds Analysis

The composition and content of ester compounds largely determined the flavor and quality of wines. Most of the esters come from the esterification reaction of alcohol and acid during fermentation. Esters were one of the dominant volatile compounds with high odor activity values in coffee pulp wines that were pivotal for desirable fruity and floral aromas in fruit wines [28]. Eighteen ethyl esters were formed in this experiment. There are five kinds of characteristic ester components, which are 3-methyl-1-butanol acetate, hexanoic acid ethyl ester, octanoic acid ethyl ester, decanoic acid ethyl ester, and acetic acid 2-phenylethyl ester.

Octanoic acid ethyl ester is the most important characteristic component in coffee pulp wines, which gives the wine body a brandy flavor and has been detected and reported in many wine bodies [29]. Hexanoic acid ethyl ester had the aroma of sweet apple and was the key main aroma of strong-flavor liquor [27]. The two constitute the main flavor of the ester aroma in coffee pulp wines and contribute to the fruit brandy-flavored wine body. Other components, such as 3-methyl-1-butanol acetate, decanoic acid ethyl ester, and acetic acid 2-phenylethyl ester, enriched the wine body and made the whole wine body softer.

#### 2.2.3. Ketone and Phenolic Compounds Analysis

β-damascenone was an important aroma component in coffee pulp wines, which endowed coffee pulp wines with strong and pleasant floral and sweet aromas. Damascenone was considered to be the key aroma of Chinese fragrance-type liquor [30]. It was derived from carotenoids and glycosides in Baijiu, which was detected and reported in wines, cider, and so on [31,32].

Phenolic components were the characteristic components of sauce-flavor liquor. A total of ten phenolic substances were detected in this experiment, among which a characteristic component was 2-methoxyphenol. According to the fermentation mechanism, 2-methoxy-phenol and its derivatives were produced by ferulic acid in the fermentation process of S. cerevisiae. Ferulic acid had two routes in coffee pulp wines. One route was converted from lignin, and the other was produced from the degradation of chlorogenic acid in coffee pulps [33]. The presence of phenolic compounds not only endowed the style and taste of coffee pulp wines but also had the function of scavenging free radicals, which could resist aging and prevent the occurrence of many diseases [24].

#### 2.2.4. Other Compounds Analysis

Two terpene compounds, linalool and citronella, were found to affect the aroma of coffee pulp wines. But they only played an important role in modifying the flavor of the coffee pulp wine (0.1 ≤ ROAV < 1). In contrast, linalool contributes slightly more to the flavor of coffee pulp wines, providing citrus, orange, and floral flavors [27]. Nonanal has little effect on the flavor of coffee pulp wine and only plays a modification role.

Six acids were detected in this experiment. The presence of acid components did not affect the aroma of coffee pulp wines, but its accumulation could have a toxic effect on the growth of S. cerevisiae [34].

### 2.3. Sensory Evaluation Profiles

#### 2.3.1. Sensory Evaluation

The sensory evaluation was completed by the sensory evaluation group. Although individuals might have personal preferences in the evaluation, the deviation was not large. The results are shown in Table 3. Sample B and sample C obtained a better score, consistent with their higher flavor component contents (Figure 1d). According to the results of the single-strain fermentation experiment, the sensory conclusion and flavor content were corresponding. Similar results were also found in this experiment except for sample E [16]. Sample E had slightly less compound content than samples B and C but had the highest sensory score (86.46 ± 0.36). However, the sensory scores of sample E and sample B were not statistically different. It was possible that sample E was prepared from four strains of S. cerevisiae (CICC1425, CICC1557, CICC1793, and CICC32762), while sample B was prepared from three of the yeasts (CICC1425, CICC1557, and CICC32762). The four yeasts influenced each other during the fermentation to give a good sensory score for sample E.

#### 2.3.2. E-Nose and E-Tongue Evaluation

**Figure 2:**
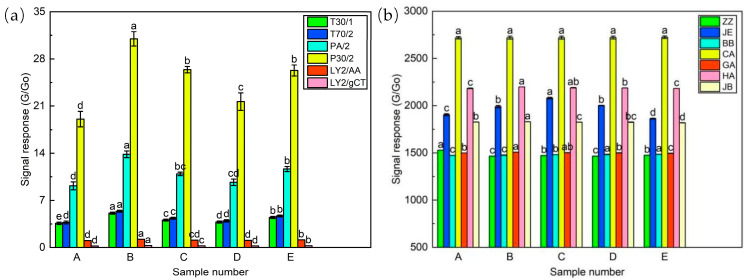
Sensor responses of E-nose (**a**) and E-tongue (**b**) for coffee pulp wines. (Different lowercase letters indicate significant differences between samples (*p* < 0.05)).

The E-nose and E-tongue are good methods for analyzing samples, because they can acquire complete information related to samples [35]. The typical responses of E-nose and E-tongue to the coffee pulp wine samples are shown in Figure 2, where G/Go was used as the response value, that is, the ratio between sample gas and pure air resistivity [36].

Figure 2a shows the characteristic signal response of the six sensors of the E-nose to the samples. The P30/2 and PA/2 exhibited a substantial response, followed by the T30/1 and T70/2, while they demonstrated nearly identical response values to the samples, whereas the LY2/AA and LY2/gCT exhibited the lowest response values to the sample. The overall response of each sensor was highest in sample B, followed by sample E and sample C. Subsequently, sample D and sample A exhibited a lower response. Sample E and sample C displayed similar trends in sensor response. Furthermore, there were significant variations in sensor response values among the samples apart from sample E.

Figure 2b shows the characteristic signal response of the sensor of E-tongue to the samples. It was evident that the seven sensors exhibited elevated response values toward the samples. Among these sensors, the CA demonstrated the highest response value, succeeded by HA, JE, and JB, while the response values of ZZ, BB, and GA were relatively low. The general trend of sensor response remained consistent across various samples. With the exception of the JE, minimal disparity was observed in the sensor response values across different samples. Notably, the response values of the CA did not exhibit significant variation among the five samples.

#### 2.3.3. OPLS-DA of Sensory, E-Nose, and E-Tongue Evaluation

**Figure 3:**
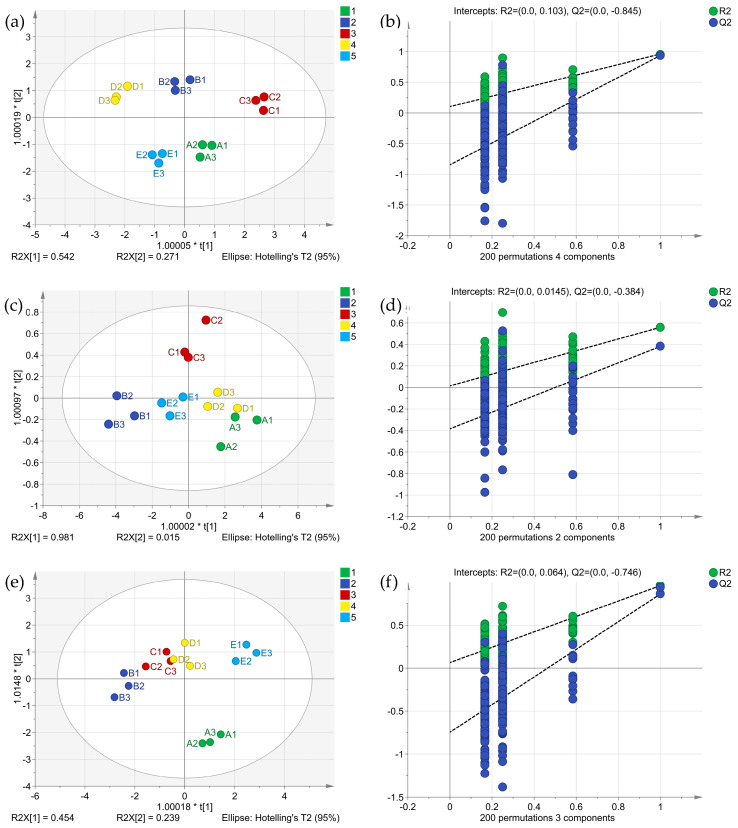
OPLS-DA analysis and model cross-validation of sensory evaluation. (**a**,**b**) are sensory evaluations, (**c**,**d**) are E-nose sensory evaluations, and (**e**,**f**) are E-tongue sensory evaluations.

For the OPLS-DA model, the degree of fit (R^2^) and predictive power (Q^2^) of the model can be used to identify coffee pulp wines [37,38]. R^2^x and R^2^y were typically used to evaluate the goodness of fit and reliability of the model, respectively. Q^2^ was typically used to evaluate the predictive ability of the model [39]. Sensory evaluation data and electronic sensory data were taken as dependent variables, and the different types of coffee wine samples were taken as independent variables. The effective differentiation of different types of samples was realized through OPLS-DA and cross-model verification. The results are shown in Figure 3.

The fitting index of the independent variable (R^2^x), the fitting index of the dependent variable (R^2^y), and the model prediction index (Q^2^) of the sensory evaluation were 0.990, 0.879, and 0.782, respectively. In theory, R^2^ and Q^2^ values close to 1 were preferable. The closer these parameters are to 1, the easier the model is to predict or interpret [40]. Generally, R^2^ and Q^2^ values greater than 0.5 were needed, and a value of more than 0.4 was acceptable [41].

The fitting index of the independent variable (R^2^x) of the E-tongue and E-nose was 0.764 and 0.896, the fitting index of the dependent variable (R^2^y) was 0.720 and 0.446, and the model prediction index (Q^2^) was 0.559 and 0.528, respectively. After 200 permutation tests, the intersection point of the Q^2^ regression line and the vertical axis was less than zero, indicating that there was no overfitting in the model and that the model verification was effective. The OPLS-DA differentiation results could potentially be used for the evaluation of coffee pulp wines.

### 2.4. Multivariate Statistical Analysis of Characteristic Compounds and Sensory Evaluation

#### 2.4.1. PCA of Characteristic Compounds

**Figure 4:**
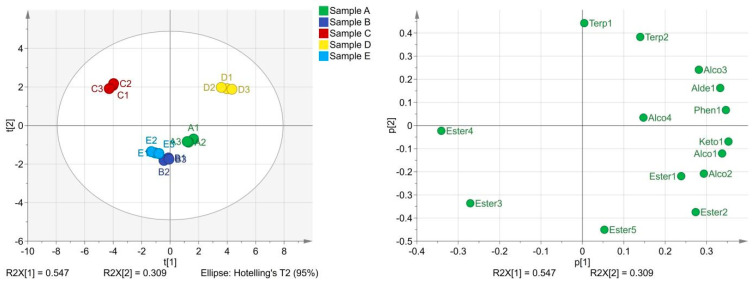
Principal component analysis diagram and load diagram of characteristic volatile components.

PCA was conducted among samples on the characteristic compounds, and the results are shown in Figure 4. The cumulative contribution rate of the first two principal components was 85.6%, which could express all the sample information, and the result was credible. The five samples of coffee pulp wines were located in three different quadrants, reflecting the difference in characteristic flavor among the samples. Sample A is located in the fourth quadrant, corresponding to six compounds with a high matching degree. They are 3-methyl-1-butanol acetate, hexanoic acid ethyl ester, acetic acid 2-phenylethyl ester, 3-methyl-1-butanol, 1-heptanol, and β-damascenone. β-damascenone was the most important characteristic component in coffee pulp wines, and hexanoic acid ethyl ester was also the key characteristic component. The other four components play an important role in modifying the flavor of coffee pulp wines.

Sample B and sample E were in the third quadrant, and octanoic acid ethyl ester and decanoic acid ethyl ester had a high matching degree with them. Octanoic acid ethyl ester was considered to be the main characteristic component, while decanoic acid ethyl ester only modifies the flavor of coffee pulp wines. Sample D was located in the first quadrant and had six characteristic components with a high matching degree. They all play an important role in modifying the flavor of coffee pulp wines. Sample C is located in the second quadrant and has no highly matched compounds.

A PCA of characteristic components verified the possibility of flavor differences caused by chemical component differences but also explained the material source for the formation of sensory flavor differences. Sample A and sample D were highly matched with six compounds, but their sensory scores were significantly different from other samples. There was no significant difference in sensory scores between sample C and sample E, but there was a significant difference between them and sample B. However, sample B and sample E are closer in the principal component analysis. This also proved that the formation of sensory quality was not determined by the number and content of flavor compounds but by the interaction with other related flavor compounds.

#### 2.4.2. Mantel Test of Sensory Evaluation and Characteristic Compounds

**Figure 5:**
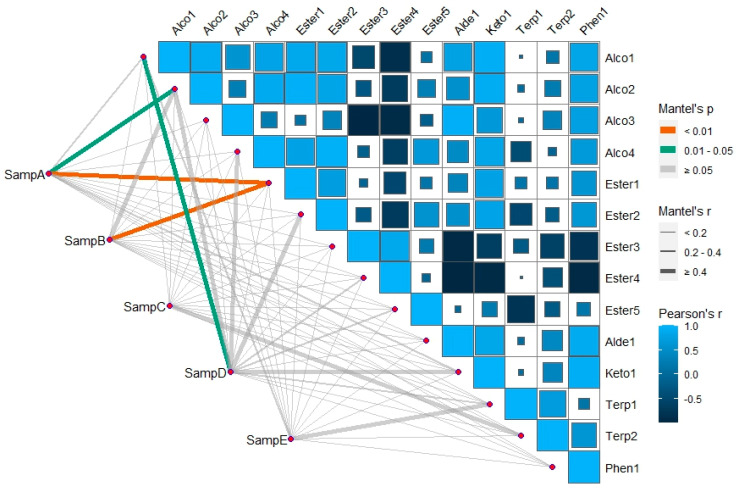
Mantel test between characteristic components and sensory evaluation. (Samp is short for sample. Capital letters A–E are sample numbers.)

A Mantel test was performed to explore the correlation between sensory evaluation and characteristic components. The data matrix of sensory evaluation related to characteristic components was used for analytical evaluation, including aroma, flavor, overall, acidity, and liquor. The results are shown in Figure 5. The correlation between the sensory evaluation data matrix and the characteristic component data matrix could be determined by observing the color and thickness of the line. A darker and thicker line indicated a stronger correlation. Additionally, it could be inferred that the sensory evaluation of samples was influenced by the coordination of various characteristic components [42].

The correlation between sensory evaluation and characteristic flavor was not very significant. The difference between sample A and 3-Methyl-1-butanol was highly significant, and that between sample A and 1-Heptanol was significant, while that between sample A and other characteristic compositions was not significant. Sample D varied significantly from 3-Methyl-1-butanol and not from other characteristic components. Neither sample C nor sample E differ significantly from the characteristic components. It may be that the sensory evaluation, although related to the characteristic flavor, was also affected by other volatile and non-volatile components, which was not exactly the same as the performance of the characteristic components.

The color gradient observed in the characteristic component data matrix suggested the presence of a correlation among the characteristic components. Specifically, lighter colors signified a positive correlation, whereas darker colors indicated a negative correlation. Additionally, the size of the square denoted the magnitude of significance [43]. Figure 5 demonstrates that the correlation between characteristic components did not necessarily correspond to its content. It was plausible that despite the lowROAV of certain components, their collaboration with other components concurrently enhances the aroma via taste. [31].

## 3. Materials and Methods

### 3.1. Preparation of Coffee Pulp Wine Samples

The experimental raw material was Arabica coffee pulp, which was collected from Munaihe Industrial Park, Simao District, Pu’er City, Yunnan Province. The sampling work was assisted by the College of Tropical Crops, Yunnan Agricultural University. Coffee pulp was prepared by wet processing of coffee cherry and contains approximately 50% pectin of the total component. The fermentation technology of coffee pulp wine adopted the previous method of the research group but was slightly modified [16].

In total, 5.0 mg coffee pulp powder and 170 mL water were precisely measured and placed into a 250 mL triangular flask. Subsequently, cellulase (0.25 g), pectinase (0.12 g), and pancreatic enzyme (0.02 g) were added simultaneously. The enzymatic digestion process was then conducted for a duration of 3 h at a temperature of 50 °C. After enzymatic digestion, granulated sugar (22.5 g) and sodium bisulfite (0.008 g) were also added to the triangular flask and sterilized at 121 °C for 20 min. After cooling, fermented liquid strain (2 mL) was added and then fermented at 28 °C for 21 days. After fermentation, the fermentation broth was centrifuged at 9000 r/min for 10 min. The 4 species of S. cerevisiae were purchased from the China Center of Industrial Culture Collection (CCIC), and their serial numbers were CICC1425, CICC1793, CICC1557, and CICC32762. The samples were named from A to E, and the strain composition patterns of the samples are shown in Table 4.

### 3.2. Volatile Component Analysis

The volatile compounds of the samples were determined by HS-SPME-GC—MS. The extraction fraction was completed by HS-SPME (PAL RSI 85, CTC Analytics AG, Zwingen, Switzerland). The serial number of the PAL 3 autosampler syringe was the Robot. The heating oscillator was numbered Agitator 1 for the heating, oscillation, and extraction of the samples, with a rotation rate of 250 r/min.

An accurately weighed 2.0 mL sample and 0.6 g sodium chloride were filled into 20 mL screw vials fitted with polytetrafluorethylene silicone septa. The samples were incubated at 50 °C for 20 min and adsorbed for 30 min with a 75 µm CAR/PDMS fiber supplied by Anpel (Anpel laboratory technologies, Shanghai, China). After extraction, the fiber was immediately inserted into the injection port of the GC—MS for the desorption step at 250 °C for 5 min [44].

GC—MS analyses were performed on an Agilent 7890A GC 5975C MS system coupled with a quadrupole mass filter for mass spectrometric detection (Agilent Technologies, Santa Clara, CA, USA). Chromatographic separation was achieved on a DB-5MS capillary column (30 m × 0.25 mm × 0.25 μm). The analytical conditions were as follows: the oven’s initial temperature was held at 50 °C for 5 min, increased to 240 °C at 5 °C min^−1^, and then further increased to 250 °C at 1 °C min^−1^. Helium was used as the carrier gas, with a flow rate of 1 mL min^−1^. The injector and transfer line temperatures were 250 °C and 280 °C, respectively. The detection was performed in full scan mode over the mass range 35–300 *m*/*z*. The integration start time was 5.00 min to avoid solvent peaks [36].

### 3.3. Identification and Quantification of Volatile Components

The qualitative method of unknown compounds was carried out by the following two methods: The first method is mass spectrometry, compared with the NIST 17 standard library. The other method is used to calculate the linear retention index (LRI), which is compared with the reference retention index (RRI). The linear retention index was detected using n-alkanes (C7-C30), which were purchased from Supelco (Sigma-Aldrich, Bellefonte, PA, USA) and calculated as follows [45]:

$$LRI=100\times n+\frac{100\times \left(t_{x}-t_{n}\right)}{t_{n+1}-t_{n}}$$

where LRI represents the linear retention index, tx represents the retention time, and tn and tn+1 represent the retention times of the n-alkanes having n and n + 1 carbon atoms, respectively, where t_n+1_ > t_x_ > t_n_.

The quantitative method was an external standard method with 3-octanol as the standard. The standard solution of different concentrations is configured with dichloromethane as the solvent to draw the standard curve of 3-octanol. The default response factor for each compound to 3-octanol is 1. It was used to calculate the content of each compound.

### 3.4. Analysis of Relative Odor Activity Values (ROAVs)

The ROAV parameter was introduced to evaluate the contribution of each volatile compound to the sample flavor properties [46]. The component that contributes the most to the flavor of the sample is defined as ROAVstan with a given value of 100, and the ROAV of the remaining volatile components is calculated according to the following equation:

$${ROAV}_{i}\approx \frac{C_{ri}}{C_{rstan}}\times \frac{T_{stan}}{T_{i}}\times 100$$

where C_ri_ and T_i_ represent the relative content and the flavor threshold of each volatile flavor compound, respectively; C_rstan_ and T_stan_ represent the relative content and the flavor threshold of the component that contributes the most to the overall odor of each sample, respectively.

### 3.5. Sensory Evaluation

The sensory evaluation was conducted by a sensory evaluation team composed of 4 men and 3 women, mainly master’s students, who were doing graduation projects in our unit. These students had undergone comprehensive and systematic curriculum learning in school, encompassing the principles and methodologies of food sensory evaluation. Prior to conducting the sensory evaluation, we also implemented flavor correction techniques to enhance the precision of the sensory assessment process. Sensory evaluation was carried out in a professional sensory evaluation room. Samples were prepared in the preparation area, and flavor calibration and sample testing were performed in the testing area.

The design of the evaluation index was completed by referring to the evaluation standards of Chinese liquor and coffee cupping, while we set 9 indices, including aroma, flavor, aftertaste, sweetness, liquor, acidity, cleanliness, balance, and overall. The overall index was 20 points, and the other indices were 10 points [47]. The total score of the sensory evaluation was 100 points. Each indicator was scored according to the intensity of the attribute.

The aroma index was determined by nose to evaluate the intensity of volatile aroma. The indexes of flavor, aftertaste, sweetness, liquor, and acidity were evaluated by the sensory intensity of coffee pulp wines in the mouth and scored according to the intensity. The cleanliness index not only evaluates whether the coffee pulp wine was pure but also determines whether there was an undesirable flavor. The balance index was for the above indicators, to evaluate the indicators in coffee pulp wines’ balance. The overall index was a special index that mainly describes the overall coordination of coffee pulp wines. A few individual preferences could be added to the overall assessment.

### 3.6. E-Tongue Evaluation

Coffee taste attributes were evaluated using an Astree II potentiometric electronic tongue (Alpha M. O. S., Toulouse, France). This E-tongue system comprises an LS48 automatic sampler, sensor array, reference electrode (Ag/AgCl), and a chemometric software package. The E-tongue contained seven chemical sensors (ZZ, JE, BB, CA, GA, HA, and JB), which are potentiometric sensors with an organic membrane coating with specific sensitivity and selectivity for each sensor. The coffee pulp wine sample was centrifuged first, and a 20 mL centrifuged sample was added to the test beaker, followed by 60 mL of ultrapure water. The parameters were a cleaning time of 10 s and a stirring speed of 1 r/s. The data were collected at 25 °C for 210 s and repeated 8 times. Between each measurement, 80 mL of deionized water was used to clean the sensors, and the sensor signals were acquired using Astree software v.3.0.1. [36].

### 3.7. E-Nose Analysis

E-nose detection uses the Gemini olfactory analysis system. The structure consists of pattern recognition software, an HS-100 autosampler, and a sensory array unit. The sensory array is equipped with six metal oxide semiconductor sensors (T30/1, T70/2, PA/2, P30/2, LY2/AA, and LY2/gCT) (Alpha M. O. S., Toulouse, France). Since the sensors cannot directly detect samples containing ethanol, it is necessary to pretreat the samples. The samples of coffee pulp wine were adsorbed by an organic phase filter membrane and kept in air for 1 h. After the ethanol was volatilized on the filter membrane, the filter membrane was loaded into the sample detection vial. The olfactory analysis system used air as the carrier gas at a flow rate of 150 mL/min. The samples were incubated before injection and incubated for 5 min at 80 °C (300 rpm). The injection volume of the sample was 1500 μL [36].

### 3.8. Statistical Analysis

The experiments were conducted in triplicate, and the experimental data were presented as the mean value accompanied by the standard deviation. Statistical analysis was performed through a one-way analysis of variance (ANOVA), followed by Duncan’s multiple test to identify significant differences (SPSS 22.0, SPSS Inc., Chicago, IL, USA). A Mantel test analysis was performed using R studio with the ggcor package (version 2022.07.2, The R Foundation for Statistical Computing, Vienna, Austria). Origin 2022 was used for data plotting (OriginLab Corporation, Northampton, MA, USA). Principal component analysis (PCA) and orthogonal partial least squares discriminate analysis (OPLS-DA) were performed using SIMCA software (version 14.1, Umetrics, Umeå, Sweden). The Venn diagram was drawn on the Evenn network platform (http://www.ehbio.com/test/Venn/#/ (accessed on 21 September 2023)).

## 4. Conclusions

The mixed fermentation method of four S. cerevisiae strains was used to produce coffee pulp wines, and the flavor and sensory characteristics were compared and evaluated. The comprehensive analysis identified that there were 87 volatile components in the five different types of coffee pulp wines, with significant variations in content among the samples, which were 45.05 mg/g, 56.80 mg/g, 51.26 mg/g, 42.97 mg/g, and 50.05 mg/g, respectively. The chemical composition of each sample was significantly different, but the proportion of common components was basically unchanged, which was mainly caused by the difference in yeast species.

Fourteen characteristic components mainly provided a sweet and floral apple brandy flavor for coffee pulp wines. However, each sample also had a unique flavor due to the content difference, which could be effectively distinguished by OPLS-DA. PCA and the Mantel test confirmed significant differences in the characteristic components associated with the sensory evaluation of the samples. These could better explain the results that produced the same characteristic components but different flavors, further explaining the difference in sensory scores between samples.

This study confirmed that coffee pulp can be used to prepare coffee pulp wine with good flavor, and also confirmed the phenomenon of flavor and taste differences caused by different strain combinations. This may be the result of several strains affecting each other during fermentation. By comprehensive comparison, the fermentation modes of four strains combined were selected as suggestions for coffee pulp wine production. Meanwhile, the trend of microflora change and metabolite generation during the fermentation process will be the next research focus.

## Figures and Tables

**Table 1:** Volatile compounds of coffee pulp wines.

RT (min)	LRI	RRI	Compounds	Relative Content (%)				
				Sample A	Sample B	Sample C	Sample D	Sample E
Alcohols								
6.01	<1100	1086	2-methyl-1-propanol	1.61 ± 0.06 ^d^	1.38 ± 0.06 ^d^	1.52 ± 0.06 ^c^	2.09 ± 0.10 ^a^	1.63 ± 0.06 ^b^
10.98	1211	1209	3-methyl-1-butanol	32.7 ± 0.47 ^a^	22.85 ± 0.29 ^e^	23.75 ± 0.22 ^d^	30.28 ± 0.24 ^b^	27.69 ± 0.33 ^c^
12.71	1242	1248	3-methyl-3-buten-1-ol	0.09 ± 0.00 ^b^	0.11 ± 0.01 ^a^	0.07 ± 0.01 ^c^	0.05 ± 0.01 ^d^	0.07 ± 0.00 ^c^
14.15	1267	1268	Acetoin	0.45 ± 0.02 ^b^	0.42 ± 0.02 ^b^	0.29 ± 0.02 ^e^	0.78 ± 0.05 ^a^	0.37 ± 0.02 ^d^
17.03	1317	1320	2-heptanol	0.05 ± 0.00 ^a^	0.03 ± 0.00 ^c^	0.03 ± 0.01 ^bc^	0.04 ± 0.01 ^b^	—
17.26	1321	1325	3-methyl-1-pentanol	0.06 ± 0.01 ^a^	0.04 ± 0.00 ^c^	0.05 ± 0.00 ^b^	—	0.05 ± 0.00 ^b^
18.82	1346	1345	1-hexanol	0.19 ± 0.01 ^a^	0.10 ± 0.00 ^d^	0.12 ± 0.01 ^c^	0.18 ± 0.01 ^a^	0.13 ± 0.01 ^b^
24.91	1451	1447	1-heptanol	0.50 ± 0.02 ^a^	0.37 ± 0.02 ^b^	0.34 ± 0.02 ^c^	0.37 ± 0.02 ^b^	0.32 ± 0.01 ^c^
26.97	1489	1484	2-ethyl-1-hexanol	0.85 ± 0.02 ^ab^	0.67 ± 0.02 ^c^	0.67 ± 0.03 ^c^	0.89 ± 0.05 ^a^	0.81 ± 0.04 ^b^
27.96	1507	1509	(S)-3-ethyl-4-methylpentanol	0.16 ± 0.01 ^a^	0.11 ± 0.01 ^c^	0.13 ± 0.01 ^b^	0.16 ± 0.01 ^a^	0.13 ± 0.01 ^b^
28.85	1522	1521	2-nonanol	0.09 ± 0.01 ^b^	0.07 ± 0.00 ^c^	0.07 ± 0.00 ^c^	0.13 ± 0.01 ^a^	0.08 ± 0.01 ^bc^
29.59	1534	1539	2,3-butanediol	—	0.09 ± 0.01 ^b^	0.10 ± 0.01 ^b^	0.28 ± 0.03 ^a^	0.30 ± 0.02 ^a^
30.92	1557	1557	1-octanol	0.12 ± 0.01 ^c^	0.10 ± 0.01 ^d^	0.15 ± 0.01 ^b^	0.22 ± 0.01 ^a^	0.11 ± 0.01 ^cd^
36.19	1648	1640	2-furanmethanol	0.18 ± 0.01 ^a^	0.15 ± 0.01 ^bc^	0.11 ± 0.01 ^d^	0.16 ± 0.01 ^b^	0.14 ± 0.01 ^c^
36.73	1658	1660	1-nonanol	0.13 ± 0.01 ^d^	0.13 ± 0.01 ^d^	0.17 ± 0.01 ^c^	0.45 ± 0.03 ^a^	0.23 ± 0.02 ^b^
44.62	1824	1820	Isogeraniol	0.05 ± 0.00 ^c^	0.06 ± 0.00 ^b^	0.07 ± 0.00 ^a^	0.08 ± 0.00 ^a^	0.05 ± 0.00 ^c^
45.50	1840	1823	2-dodecanol	—	—	0.17 ± 0.01 ^a^	0.04 ± 0.00 ^b^	—
47.22	1871	1870	Benzyl alcohol	0.52 ± 0.04 ^c^	0.58 ± 0.03 ^b^	0.66 ± 0.03 ^a^	0.47 ± 0.02 ^d^	0.50 ± 0.02 ^c^
49.01	1904	1906	Phenylethyl alcohol	24.44 ± 0.23 ^b^	25.26 ± 0.22 ^a^	23.17 ± 0.22 ^d^	20.32 ± 0.06 ^e^	23.66 ± 0.10 ^c^
52.03	1985	1966	1-dodecanol	0.10 ± 0.01 ^e^	0.31 ± 0.02 ^c^	0.79 ± 0.04 ^a^	0.36 ± 0.02 ^b^	0.16 ± 0.01 ^d^
57.47	2309	—	Diglycerol	—	—	—	—	0.06 ± 0.00 ^a^
43.26	1800	1800	7-methyl-3-methylene-6-octen-1-ol	—	0.16 ± 0.01 ^c^	0.24 ± 0.02 ^a^	0.22 ± 0.01 ^b^	0.24 ± 0.01 ^a^
Esters								
6.89	1120	1122	3-methyl-1-butanol, acetate	0.85 ± 0.03 ^a^	0.51 ± 0.04 ^b^	0.51 ± 0.04 ^b^	0.51 ± 0.04 ^b^	0.51 ± 0.03 ^b^
7.38	1131	1134	Pentanoic acid, ethyl ester	0.07 ± 0.00 ^a^	0.05 ± 0.00 ^b^	0.02 ± 0.00 ^e^	0.04 ± 0.00 ^c^	0.03 ± 0.00 ^d^
12.00	1229	1233	Hexanoic acid, ethyl ester	5.24 ± 0.05 ^d^	5.34 ± 0.06 ^c^	6.33 ± 0.08 ^a^	4.70 ± 0.07 ^e^	5.77 ± 0.09 ^b^
15.15	1285	—	(E)-hex-4-enoic acid ethyl ester	9.52 ± 0.44 ^d^	13.44 ± 0.33 ^a^	12.01 ± 0.37 ^c^	12.13 ± 0.25 ^c^	12.49 ± 0.22 ^b^
15.85	1298	1292	(Z)-3-hexenoic acid, ethyl ester	0.37 ± 0.02 ^c^	0.41 ± 0.02 ^b^	0.23 ± 0.02 ^d^	0.47 ± 0.02 ^a^	0.23 ± 0.02 ^d^
17.49	1324	1331	Heptanoic acid, ethyl ester	0.19 ± 0.01 ^b^	0.17 ± 0.01 ^c^	0.30 ± 0.01 ^a^	0.19 ± 0.01 ^b^	0.19 ± 0.01 ^b^
22.00	1397	—	(Z)-3-hexenoic acid, butyl ester	—	0.04 ± 0.00 ^a^	0.04 ± 0.00 ^a^	—	0.04 ± 0.00 ^a^
23.48	1424	1429	Octanoic acid, ethyl ester	2.38 ± 0.15 ^c^	2.49 ± 0.14 ^c^	4.91 ± 0.28 ^a^	1.66 ± 0.06 ^c^	2.72 ± 0.13 ^b^
23.89	1432	1435	Methyl sorbate	0.12 ± 0.01 ^b^	0.15 ± 0.01 ^a^	0.12 ± 0.01 ^b^	0.12 ± 0.01 ^b^	0.10 ± 0.01 ^c^
25.77	1467	—	4-octenoic acid, ethyl ether	—	—	0.04 ± 0.00 ^a^	—	—
25.94	1470	—	2,4-hexadienoic acid, ethyl ester	0.43 ± 0.04 ^d^	0.72 ± 0.04 ^a^	0.58 ± 0.03 ^c^	0.60 ± 0.04 ^bc^	0.62 ± 0.05 ^b^
26.39	1479	1471	7-octenoic acid, ethyl ester	0.16 ± 0.01 ^b^	0.13 ± 0.01 ^c^	0.20 ± 0.02 ^a^	0.10 ± 0.00 ^d^	0.14 ± 0.01 ^c^
27.29	1495	1501	(E,E)-2,4-hexadienoic acid, ethyl ester	2.42 ± 0.12 ^d^	6.01 ± 0.25 ^a^	4.07 ± 0.19 ^c^	4.36 ± 0.20 ^b^	4.08 ± 0.13 ^c^
29.47	1532	1531	Nonanoic acid, ethyl ester	0.05 ± 0.00 ^b^	0.05 ± 0.00 ^b^	0.08 ± 0.00 ^a^	—	—
35.32	1633	1638	Decanoic acid, ethyl ester	0.94 ± 0.04 ^c^	1.06 ± 0.04 ^b^	2.34 ± 0.16 ^a^	0.72 ± 0.06 ^d^	1.15 ± 0.08 ^b^
36.43	1653	1651	Octanoic acid, 3-methylbutyl ester	0.11 ± 0.01 ^b^	0.11 ± 0.01 ^b^	0.20 ± 0.01 ^a^	0.08 ± 0.00 ^c^	0.11 ± 0.00 ^b^
38.11	1683	1696	Ethyl 9-decenoate	0.25 ± 0.02 ^c^	0.28 ± 0.02 ^bc^	0.75 ± 0.05 ^a^	0.13 ± 0.00 ^d^	0.29 ± 0.01 ^b^
42.54	1783	1783	Benzeneacetic acid, ethyl ester	0.08 ± 0.00 ^b^	0.08 ± 0.00 ^b^	0.10 ± 0.01 ^a^	0.11 ± 0.00 ^a^	0.08 ± 0.00 ^b^
44.02	1814	1813	Acetic acid, 2-phenylethyl ester	0.96 ± 0.06 ^c^	1.58 ± 0.11 ^a^	1.47 ± 0.10 ^a^	0.84 ± 0.06 ^d^	1.18 ± 0.07 ^b^
46.36	1855	1841	Dodecanoic acid, ethyl ester	0.91 ± 0.05 ^b^	1.03 ± 0.05 ^a^	1.11 ± 0.07 ^a^	0.92 ± 0.07 ^b^	0.92 ± 0.07 ^b^
48.54	1894	1892	Lauryl acetate	—	0.07 ± 0.00 ^b^	0.10 ± 0.00 ^a^	0.11 ± 0.01 ^a^	0.07 ± 0.00 ^b^
52.84	2014	2024	γ-nonalacton	0.25 ± 0.01 ^a^	0.26 ± 0.01 ^a^	0.23 ± 0.02 ^b^	0.27 ± 0.02 ^a^	0.18 ± 0.01 ^c^
53.89	2071	2049	Tetradecanoic acid, ethyl ester	0.05 ± 0.00 ^b^	0.05 ± 0.00 ^b^	0.06 ± 0.00 ^a^	0.05 ± 0.00 ^b^	0.05 ± 0.00 ^b^
55.7	2157	2160	Hexanoic acid, 2-phenylethyl ester	0.11 ± 0.00 ^c^	0.16 ± 0.01 ^a^	0.06 ± 0.01 ^d^	0.12 ± 0.01 ^bc^	0.13 ± 0.00 ^b^
56.98	2247	2251	Hexadecanoic acid, ethyl ester	0.19 ± 0.01 ^a^	0.15 ± 0.01 ^b^	0.12 ± 0.01 ^d^	0.15 ± 0.01 ^b^	0.13 ± 0.00 ^c^
Acids								
19.10	1350	—	1,2-dimethyl-cyclopent-2-enecarboxylic acid	0.06 ± 0.00 ^b^	0.07 ± 0.00 ^a^	—	0.07 ± 0.00 ^a^	0.06 ± 0.00 ^b^
24.21	1438	1436	Acetic acid	0.24 ± 0.02 ^a^	0.21 ± 0.01 ^b^	0.13 ± 0.01 ^d^	0.25 ± 0.02 ^a^	0.16 ± 0.01 ^c^
31.19	1561	—	2-methyl-propanoic acid	0.10 ± 0.01 ^d^	0.11 ± 0.01 ^c^	0.09 ± 0.01 ^d^	0.19 ± 0.01 ^a^	0.13 ± 0.01 ^b^
54.07	2081	2075	Octanoic acid	0.79 ± 0.06 ^c^	1.15 ± 0.07 ^a^	0.81 ± 0.04 ^bc^	0.40 ± 0.02 ^d^	0.87 ± 0.04 ^b^
50.11	1934	1930	(E)-3-hexenoic acid	0.34 ± 0.02 ^c^	0.52 ± 0.02 ^a^	0.32 ± 0.03 ^c^	0.52 ± 0.04 ^a^	0.46 ± 0.03 ^b^
57.20	2281	2276	n-decanoic acid	0.45 ± 0.03 ^d^	0.80 ± 0.04 ^a^	0.59 ± 0.03 ^b^	0.38 ± 0.02 ^e^	0.51 ± 0.03 ^c^
Aldehydes								
20.73	1377	1380	Nonanal	0.20 ± 0.01 ^b^	0.16 ± 0.01 ^c^	0.14 ± 0.01 ^d^	0.54 ± 0.03 ^a^	0.20 ± 0.01 ^b^
24.37	1441	1432	Furfural	0.18 ± 0.01 ^a^	0.15 ± 0.01 ^b^	0.09 ± 0.00 ^d^	0.16 ± 0.01 ^b^	0.11 ± 0.00 ^c^
27.45	1498	1502	Benzaldehyde	5.85 ± 0.23 ^c^	5.89 ± 0.28 ^c^	3.42 ± 0.22 ^d^	7.33 ± 0.43 ^a^	6.26 ± 0.31 ^b^
Ketones								
9.30	1175	1173	2-heptanone	0.09 ± 0.00 ^c^	0.11 ± 0.01 ^b^	0.13 ± 0.01 ^a^	0.10 ± 0.00 ^b^	0.05 ± 0.00 ^d^
20.49	1373	1379	2-nonanone	0.28 ± 0.02 ^b^	0.25 ± 0.01 ^c^	0.30 ± 0.01 ^a^	0.32 ± 0.03 ^a^	0.27 ± 0.02 ^b^
26.71	1485	1479	1-(2-furanyl)-ethanone	0.06 ± 0.00 ^a^	0.05 ± 0.00 ^b^	—	—	—
34.66	1621	1627	Acetophenone	0.11 ± 0.00 ^a^	0.09 ± 0.01 ^c^	0.10 ± 0.00 ^b^	0.10 ± 0.00 ^b^	0.09 ± 0.01 ^c^
44.23	1817	1814	β-damascenone	0.11 ± 0.00 ^a^	0.09 ± 0.00 ^b^	0.11 ± 0.01 ^a^	0.11 ± 0.00 ^a^	0.09 ± 0.01 ^c^
55.19	2134	2131	6,10,14-trimethyl-2-pentadecanone	0.05 ± 0.00 ^b^	0.05 ± 0.00 ^b^	0.22 ± 0.01 ^a^	0.04 ± 0.00 ^c^	0.04 ± 0.00 ^c^
Terpenes								
25.29	1458	1451	Linalool oxide	0.11 ± 0.01 ^a^	0.09 ± 0.01 ^b^	0.08 ± 0.00 ^b^	0.06 ± 0.01 ^c^	0.06 ± 0.00 ^c^
30.31	1546	1547	Linalool	0.21 ± 0.01 ^b^	0.15 ± 0.01 ^d^	0.45 ± 0.02 ^a^	0.18 ± 0.01 ^c^	0.16 ± 0.01 ^d^
40.48	1734	1717	β-selinene	0.11 ± 0.00 ^b^	0.13 ± 0.01 ^a^	0.06 ± 0.00 ^c^	0.17 ± 0.01 ^a^	0.10 ± 0.01 ^b^
41.34	1754	1753	Methyl salicylate	0.52 ± 0.04 b	0.43 ± 0.02 b	0.57 ± 0.04 a	0.53 ± 0.02 b	0.45 ± 0.04 b
42.40	1779	1765	Citronellol	0.26 ± 0.03 ^c^	0.24 ± 0.03 ^c^	0.61 ± 0.02 ^a^	0.35 ± 0.03 ^b^	0.21 ± 0.01 ^d^
53.69	2060	2042	Nerolidol	0.06 ± 0.00 ^c^	0.07 ± 0.00 ^b^	0.09 ± 0.00 ^a^	0.08 ± 0.01 ^b^	0.07 ± 0.00 ^b^
58.04	2347	2356	Trans-farnesol	0.07 ± 0.00 ^c^	0.08 ± 0.00 ^b^	0.06 ± 0.00 ^d^	0.10 ± 0.01 ^a^	0.05 ± 0.00 ^e^
Phenols								
45.94	1848	1828	(1-butylheptyl)-benzene	—	—	0.07 ± 0.00 ^a^	—	—
46.21	1853	1858	2-methoxy-phenol	0.26 ± 0.01 ^b^	0.24 ± 0.02 ^b^	0.16 ± 0.01 ^c^	0.35 ± 0.02 ^a^	0.13 ± 0.01 ^d^
46.67	1861	—	(1-propyloctyl)-benzene	—	—	0.06 ± 0.01 ^a^	—	—
47.99	1884	1873	(1-ethylnonyl)-benzene	—	—	0.04 ± 0.00 ^a^	—	—
52.53	1999	2000	Phenol	0.05 ± 0.00 ^a^	0.04 ± 0.00 ^b^	0.05 ± 0.01 ^a^	0.05 ± 0.00 ^a^	0.03 ± 0.00 ^c^
53.31	2039	2027	4-ethenyl-1,2-dimethoxy-benzene	0.10 ± 0.00 ^a^	0.07 ± 0.00 ^b^	0.07 ± 0.00 ^b^	0.06 ± 0.00 ^c^	0.06 ± 0.00 ^c^
53.44	2046	—	(1-pentyloctyl)-benzene	—	0.04 ± 0.00 ^b^	0.06 ± 0.00 ^a^	0.03 ± 0.00 ^c^	0.03 ± 0.00 ^c^
54.33	2095	2091	3-methyl-phenol	0.09 ± 0.00 ^b^	0.11 ± 0.00 ^a^	0.09 ± 0.00 ^b^	0.06 ± 0.00 ^c^	0.09 ± 0.00 ^b^
55.98	2170	2188	2-methoxy-4-vinylphenol	—	0.06 ± 0.00 ^b^	0.16 ± 0.01 ^a^	0.02 ± 0.00 ^d^	0.05 ± 0.00 ^c^
57.54	2313	2318	2,4-di-tert-butylphenol	0.30 ± 0.01 ^a^	0.25 ± 0.01 ^b^	0.06 ± 0.00 ^d^	0.14 ± 0.00 ^c^	0.05 ± 0.00 ^e^

Note: Different lowercase letters in the same line indicate significant differences between samples (*p* < 0.05); “—” indicates that the compound is not detected.

**Table 2:** Relative odor activity values (ROAVs) and odor characteristics of volatile compounds of coffee pulp wines.

Code	Compounds	Threshold (µg/g)	Odor Description	ROAV				
				Sample A	Sample B	Sample C	Sample D	Sample E
Alco1	3-Methyl-1-butanol	250	Brandy, malty, pungent, fruity	0.56 ± 0.04 ^a^	0.37 ± 0.02 ^c^	0.20 ± 0.01 ^d^	0.55 ± 0.03 ^a^	0.41 ± 0.02 ^b^
Alco2	1-Heptanol	3	Fatty, wine	0.70 ± 0.04 ^a^	0.50 ± 0.02 ^c^	0.23 ± 0.01 ^e^	0.56 ± 0.04 ^b^	0.39 ± 0.02 ^d^
Alco3	1-Nonanol	2	Rose, orange fatty, fruity	0.27 ± 0.01 ^c^	0.26 ± 0.01 ^c^	0.17 ± 0.00 ^d^	1.03 ± 0.04 ^a^	0.43 ± 0.02 ^b^
Alco4	Phenylethyl alcohol	45	Honey, spice, rose	2.31 ± 0.05 ^a^	2.28 ± 0.09 ^a^	1.07 ± 0.06 ^c^	2.06 ± 0.12 ^b^	1.96 ± 0.06 ^b^
Ester1	3-Methyl-1-butanol, acetate	3	Fruity	1.21 ± 0.02 ^a^	0.69 ± 0.01 ^c^	0.35 ± 0.01 ^e^	0.77 ± 0.02 ^b^	0.63 ± 0.02 ^d^
Ester2	Hexanoic acid, ethyl ester	0.5	Sweet, apple	44.49 ± 0.52 ^a^	43.28 ± 0.29 ^b^	26.37 ± 0.22 ^d^	42.90 ± 0.31 ^c^	42.39 ± 0.23 ^c^
Ester3	Octanoic acid, ethyl ester	0.1	Brandy, apple	100.00 ± 0.05 ^b^	100.00 ± 0.08 ^b^	100.00 ± 0.00 ^a^	75.61 ± 1.21 ^c^	100.00 ± 0.00 ^a^
Ester4	Decanoic acid, ethyl ester	20	Coconut	0.20 ± 0.01 ^b^	0.21 ± 0.01 ^b^	0.24 ± 0.01 ^a^	0.16 ± 0.00 ^c^	0.21 ± 0.01 ^b^
Ester5	Acetic acid, 2-phenylethyl ester	20	Rose, honey, apple-like	0.20 ± 0.00 ^c^	0.32 ± 0.01 ^a^	0.15 ± 0.00 ^d^	0.19 ± 0.01 ^c^	0.22 ± 0.01 ^b^
Alde1	Nonanal	3.5	Waxy, aldehydic, citrus, floral	0.25 ± 0.01 ^b^	0.18 ± 0.00 ^d^	—	0.71 ± 0.04 ^a^	0.21 ± 0.01 ^c^
Keto1	β-Damascenone	0.00495	Floral, sweet	90.25 ± 2.25 ^b^	76.32 ± 1.96 ^c^	45.53 ± 1.05 ^e^	98.37 ± 1.79 ^a^	65.38 ± 1.62 ^d^
Terp1	Linalool	1.5	Citrus, orange, floral, waxy rose	0.60 ± 0.02 ^a^	0.41 ± 0.02 ^c^	0.63 ± 0.03 ^a^	0.55 ± 0.02 ^b^	0.39 ± 0.01 ^c^
Terp2	Citronellol	10	Rose	0.11 ± 0.00 ^bc^	0.10 ± 0.00 ^c^	0.13 ± 0.00 ^b^	0.16 ± 0.00 ^a^	—
Phen1	2-Methoxy-phenol	0.17	Smoky	6.37 ± 0.34 ^b^	5.75 ± 0.28 ^c^	1.95 ± 0.09 ^e^	9.37 ± 0.41 ^a^	2.93 ± 0.15 ^d^

Note: Different lowercase letters in the same line indicate significant differences between samples (*p* < 0.05); “—” represents that the ROAV is lower than 0.10.

**Table 3:** The sensory evaluation results.

Sensory Index	Sample Number				
	Sample A	Sample B	Sample C	Sample D	Sample E
aroma	91.11 ± 2.09 ^a^	87.38 ± 1.53 ^b^	89.01 ± 0.58 ^ab^	76.53 ± 0.95 ^c^	91.50 ± 0.89 ^a^
flavor	85.63 ± 1.15 ^b^	81.85 ± 0.64 ^c^	90.50 ± 1.16 ^a^	74.97 ± 0.40 ^d^	83.43 ± 1.02 ^c^
aftertaste	74.39 ± 0.93 ^d^	87.57 ± 1.45 ^b^	92.39 ± 1.13 ^a^	83.48 ± 0.67 ^c^	90.25 ± 0.78 ^a^
sweetness	82.18 ± 2.23 ^a^	81.55 ± 1.84 ^a^	81.43 ± 0.73 ^a^	70.36 ± 0.81 ^b^	83.10 ± 0.87 ^a^
liquor	86.57 ± 3.77 ^a^	81.93 ± 1.14 ^b^	88.77 ± 0.78 ^a^	76.54 ± 0.66 ^c^	90.52 ± 0.70 ^a^
acidity	85.16 ± 1.74 ^ab^	81.45 ± 1.03 ^b^	82.94 ± 1.04 ^ab^	75.19 ± 0.61 ^c^	83.49 ± 1.11 ^a^
cleanliness	71.08 ± 0.62 ^c^	80.41 ± 2.28 ^a^	70.37 ± 0.62 ^c^	76.73 ± 0.92 ^b^	77.22 ± 1.33 ^b^
balance	76.31 ± 0.80 ^d^	88.40 ± 1.31 ^b^	90.66 ± 0.37 ^a^	82.41 ± 0.80 ^c^	92.31 ± 0.72 ^a^
overall	83.29 ± 1.44 ^b^	81.72 ± 0.47 ^bc^	87.78 ± 0.75 ^a^	80.01 ± 0.30 ^c^	86.37 ± 0.95 ^a^
total points	81.90 ± 0.15 ^c^	83.40 ± 0.73 ^b^	86.16 ± 0.13 ^a^	77.62 ± 0.33 ^d^	86.46 ± 0.36 ^a^

Note: Different lowercase letters in the same line indicate significant differences between samples (*p* < 0.05).

**Table 4:** Yeast composition of coffee pulp wine samples.

Number	Yeast Composition
Sample A	CICC1425, CICC1557, CICC1793
Sample B	CICC1425, CICC1557, CICC32762
Sample C	CICC1425, CICC1793, CICC32762
Sample D	CICC1557, CICC1793, CICC32762
Sample E	CICC1425, CICC1557, CICC1793, CICC32762

## Data Availability

Data are contained within the article.
